# Evidence for a functional interaction of *WNT10A* and EBF1 in male-pattern baldness

**DOI:** 10.1371/journal.pone.0256846

**Published:** 2021-09-10

**Authors:** Lara M. Hochfeld, Marta Bertolini, David Broadley, Natalia V. Botchkareva, Regina C. Betz, Susanne Schoch, Markus M. Nöthen, Stefanie Heilmann-Heimbach

**Affiliations:** 1 Institute of Human Genetics, University of Bonn, School of Medicine & University Hospital Bonn, Bonn, Germany; 2 Monasterium Laboratory, Skin and Hair Research Solutions GmbH, Münster, Germany; 3 Centre for Skin Sciences, Faculty of Life Sciences, University of Bradford, Bradford, England, United Kingdom; 4 Department of Neuropathology, University of Bonn Medical Center, Bonn, Germany; University of Iceland, ICELAND

## Abstract

More than 300 genetic risk loci have been identified for male pattern baldness (MPB) but little is known about the exact molecular mechanisms through which the associated variants exert their effects on MPB pathophysiology. Here, we aimed at further elucidating the regulatory architecture of the MPB risk locus on chromosome (chr.) 2q35, where we have previously reported a regulatory effect of the MPB lead variant on the expression of *WNT10A*. A HaploReg database research for regulatory annotations revealed that the association signal at 2q35 maps to a binding site for the transcription factor EBF1, whose gene is located at a second MPB risk locus on chr. 5q33.3. To investigate a potential interaction between EBF1 and *WNT10A* during MPB development, we performed *in vitro* luciferase reporter assays as well as expression analyses and immunofluorescence co-stainings in microdissected human hair follicles. Our experiments confirm that EBF1 activates the *WNT10A* promoter and that the *WNT10A*/EBF1 interaction is impacted by the allelic expression of the MPB risk allele at 2q35. Expression analyses across different hair cycle phases and immunhistochemical (co)stainings against WNT10A and EBF1 suggest a predominant relevance of EBF1/*WNT10A* interaction for hair shaft formation during anagen. Based on these findings we suggest a functional mechanism at the 2q35 risk locus for MPB, where an MPB-risk allele associated reduction in *WNT10A* promoter activation via EBF1 results in a decrease in *WNT10A* expression that eventually results in anagen shortening, that is frequently observed in MPB affected hair follicles. To our knowledge, this study is the first follow-up study on MPB that proves functional interaction between two MPB risk loci and sheds light on the underlying pathophysiological mechanism at these loci.

## Introduction

Male-pattern baldness (MPB) is the most common form of heritable hair loss in men. The phenotype is characterized by an androgen-dependent progressive hair loss in distinct areas of the scalp. While the pathophysiological signs have been well described (reviewed elsewhere [1]), the molecular mechanisms that underlie the patterned hair loss remain elusive. Over the past decade genome-wide association studies (GWAS) have proven to be a powerful tool in the identification of contributing genetic risk factors [2, 3]. So far, 375 risk loci with over 650 independent association signals have been identified [3–12]. Despite this considerable progress in the identification of the underlying genetic factors, there is still limited functional evidence that links the identified risk variants to biological mechanisms. A deeper understanding of the molecular mechanisms at the level of individual risk loci and across risk loci is however necessary to understand the precise pathomechanisms that drive the hair loss. While several of the MPB associated loci map in the vicinity of, or even span plausible candidate genes, the majority of MPB lead variants are located in non-coding regions of the genome. Research suggests that these variants exert a regulatory effect on the expression of pathobiologically relevant genes, e.g. through modification of enhancer/promoter function or modification of transcription factor binding sites [13–15].

In 2013, our follow-up analysis of candidate single-nucleotide polymorphisms (SNPs) from a European meta-analysis on MPB identified four novel genetic risk loci. Among them an MPB risk locus on chromosome (chr.) 2q35 that was tagged by rs7349332 (*P*_*combined*_ = 3.55×10^−15^, effect allele (EA) = T, odds ratio (OR) = 1.34, 95% confidence interval (CI) = [1.27–1.42]) [8]. The association peak a this locus mapped to *WNT10A* (Wingless-Type MMTV Integration Site Family, Member 10A, ENSG00000135925, OMIM: 606268) [8] and the signal has since been consistently replicated in larger GWAS in the European population [3, 9–12]. Moreover, the 2q35 locus was the first genetic risk locus for MPB, where an allele-specific regulatory effect of the lead variant on the expression of a plausible candidate gene could be proven. An mRNA expression analysis revealed a significantly decreased expression of *WNT10A* in hair follicles of MPB risk allele carriers (rs7349332-T) that is likely to contribute to the delayed telogen to anagen transition and anagen shortening that are characteristic for MPB affected hair follicles [8, 16, 17]. However, the underlying molecular mechanism and the relevant hair follicle compartment have remained elusive.

Therefore, we aimed at further elucidating the underlying biological mechanism at the 2q35 risk locus and its role in MPB by means of *in silico* and *in vitro* experiments (S1 Fig). To this end we performed comprehensive database research, followed by cell culture-based luciferase reporter gene assays and immunofluorescence analyses. Our present study resulted in the identification of an MPB-associated variant (rs3856551-C/T, minor allele frequency (MAF) Europeans = 0.12) that is in strong linkage disequilibrium (LD) with the initially identified lead variant rs7349332 in the European population and that is located within a binding site for the transcription factor EBF1 (early B-cell factor). The latter being encoded by the *EBF1* gene, which is located at a second MPB risk locus on chr. 5q33.3. Our luciferase reporter assays show that the *WNT10A* promoter is activated upon EBF1 stimulation and that this activation is MPB risk allele dependent. To investigate the cellular (co)localization of EBF1 and WNT10A across hair follicle compartments we performed immunofluorescence stainings in microdissected human scalp hair follicles. We demonstrate that WNT10A and EBF1 are expressed in the same human hair follicle compartments suggesting a co-expression *in vivo*. Together, our data provide not only novel insights into the regulatory architecture of the 2q35 risk locus but also yield the first functional evidence for a regulatory interaction between candidate genes at different MPB risk loci.

## Results

### Database research maps 2q35 risk variant to an EBF1 binding site

To gain deeper insights into the regulatory architecture of the 2q35 risk locus, we queried the HaploReg database (v4.1) to explore known regulatory annotations in LD with rs7349332 (*r*^2^ >0.6). The database research identified rs3856551-C/T, a second MPB associated variant located 10.2 kb upstream of and in strong LD with rs7349332 (*r*^2^ = 0.96; *D*´ = 0.99). According to HaploReg annotations, rs3856551 is located intronically in *WNT10A* and within a transcription factor binding site for EBF1 (ENSG00000164330, OMIM: 164343, Fig 1A). Remarkably, the gene encoding for EBF1 is spanned by another MPB risk locus on chr. 5q33.3 (*P*_*combined*_ = 2.12×10^−11^, EA = G, OR = 0.84, 95% CI = [0.79–0.89]) [8]. This led us to hypothesize (i) that EBF1 is a regulator of *WNT10A* in MPB development and (ii) that allele-specific differences in the binding affinity of EBF1 to its target site at 2q35 explain the difference in *WNT10A* expression in hair follicles of MPB risk allele carriers for rs7349332/rs3856551.

**Fig 1:**
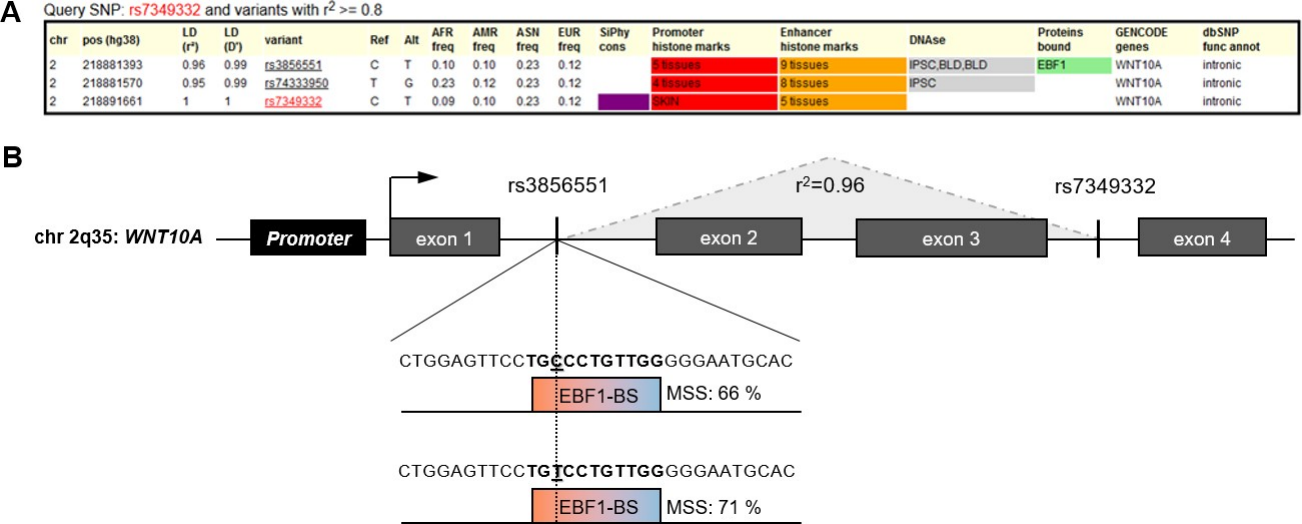
Results of the HaploReg query and genomic architecture of the 2q35 risk locus. (A) Part of the HaploReg result for rs7349332. (B) The putative *WNT10A* promoter element (1,641 bp) is located 434 bp upstream of the *WNT10A* start codon. Rs7349332, located in the third intron of *WNT10A*, is in high linkage disequilibrium (LD, *r*^2^ = 0.96) with rs3856551 located in the first intron of *WNT10A* and within an annotated EBF1 binding site (BS). Computational *in silico* analyses predicted a motif similarity score (MSS) of 66% for the MPB non-risk allele (rs3856551-C) and 71% for the risk allele rs3856551-T, suggesting higher binding affinity of EBF1 with the risk allele.

Our subsequent *in silico* prediction using position-specific-scoring matrices for putative EBF1 binding sites for the HaploReg annotated EBF1 binding site revealed that the rs3856551 MPB risk allele (T) exhibits a slightly higher binding affinity (motif similarity score [MSS] = 71%) compared to the non-risk allele (C, MSS = 66%) (Fig 1B). Based on these *in silico* data, we assumed that rs3856551 is the functionally relevant SNP at the *WNT10A* locus. Thus, rs3856551 was chosen for further functional follow-up via luciferase reporter gene assays. A HaploReg query for the MPB associated variants at the 5q33.3 locus did not identify any functional annotations.

### Luciferase reporter gene assays confirm allele-specific activation of WNT10A via EBF1

**Fig 2:**
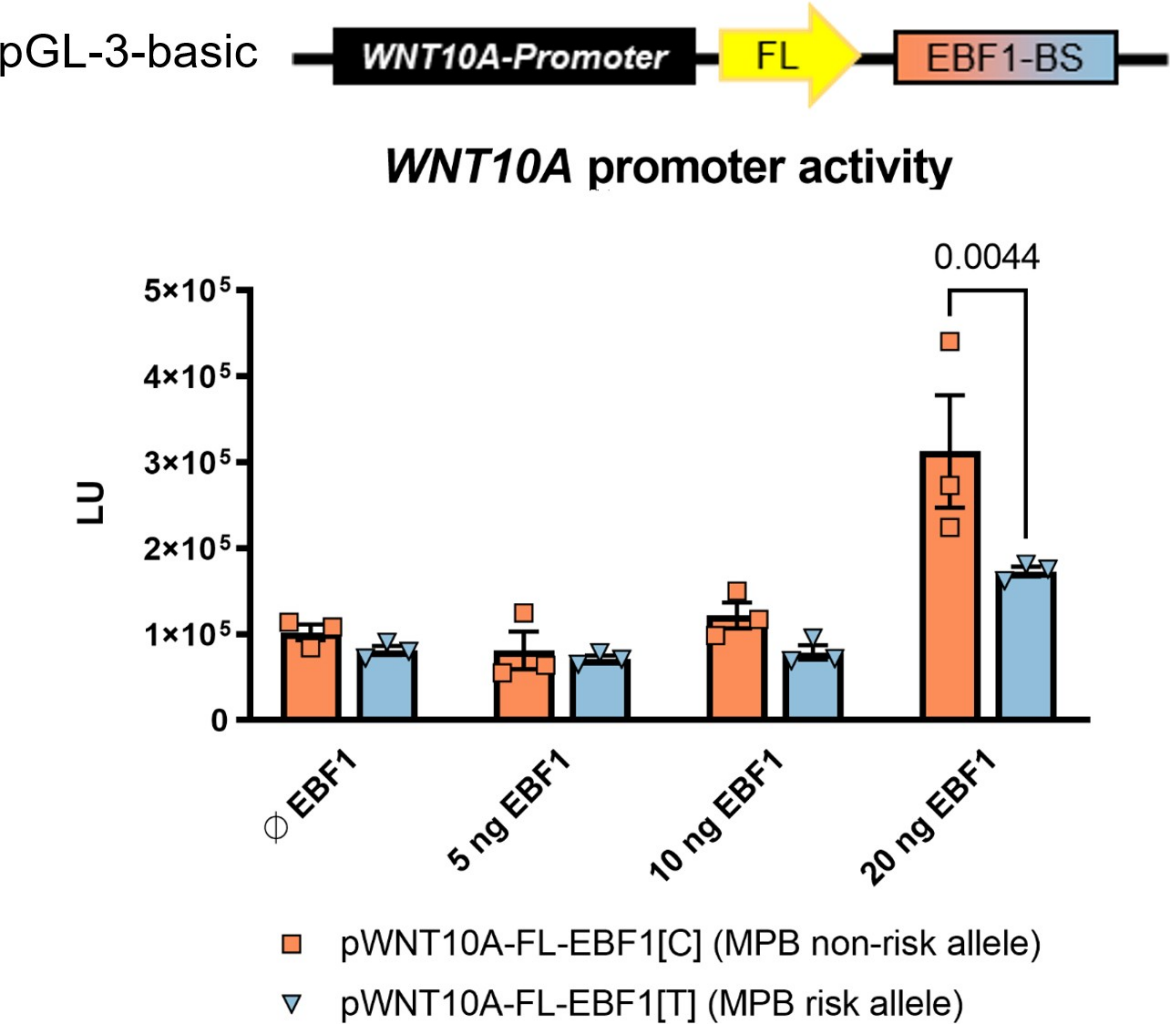
*WNT10A* promoter activity with respect to EBF1 and rs3856551. HEK-293T cells were co-transfected with the experimental reporters pWNT10A-FL-EBF1[C] (coral bars, non-risk allele-C) and pWNT10A-FL-EBF1[T] (blue bars, risk allele-T), respectively, and different concentrations of the EBF1 expression vector (pCMV-EBF1, EBF1). Five biological replicates for each construct were performed and *WNT10A* promoter activity was measured. This figure is representative for one of the five experiments in HEK-293T cells. The results for the remaining four independent experiments are shown in S2 Fig. The data represent the mean values ± SEM measured in the triplicate for each construct and experiment. *P* value was calculated with ANOVA and Tukey test. BS—binding site, FL—*Firefly*, LU—luminescence units.

To investigate whether (i) EBF1 acts as a regulator of *WNT10A* expression and whether (ii) this interaction is influenced by the 2q35 MPB risk allele, we performed luciferase reporter assays. To this end, we transiently co-transfected HEK-293T cells with (i) luciferase vectors that contained a predicted *WNT10A* promoter sequence as well as the 2q35-EBF1 binding site, harboring either the MPB risk (rs3856551-T) or the alternate allele (rs3856551-C); or the empty reporter vector pGL-3-basic as a control and (ii) an *EBF1* expression vector. Here, the sequence of the functional elements within the luciferase vectors (pWNT10A-FL-EBF1) was specifically designed to reflect the genomic architecture at the 2q35 risk locus (Figs 1 and 2). Five individual experiments for each construct were performed and *WNT10A* promoter activity was measured in triplicates for each construct and experiment. Overexpression of EBF1 did not affect the basal *Firefly* luminescence (pGL-3-basic, see S1 Table and S2 Fig). Compared to unstimulated controls (Ø EBF1), the *WNT10A*-promoter activity increased proportionally (0.9–7.2 fold) with increasing amounts of transfected EBF1 vector (5–20 ng) across all experiments. Without high EBF1 stimulation (<20 ng), no significant differences in promoter activity were observed, however stimulation at the highest EBF1 concentration (20 ng) consistently resulted in a significant (*P*<0.05) preferential promoter activation with the *WNT10A* promoter carrying the MPB non-risk allele (pWNT10A-FL-EBF1[C], Fig 2 and S2 Fig, 20 ng EBF1).

### WNT10A and EBF1 are co-expressed across hair cycle stages and within hair follicle compartments

**Fig 3:**
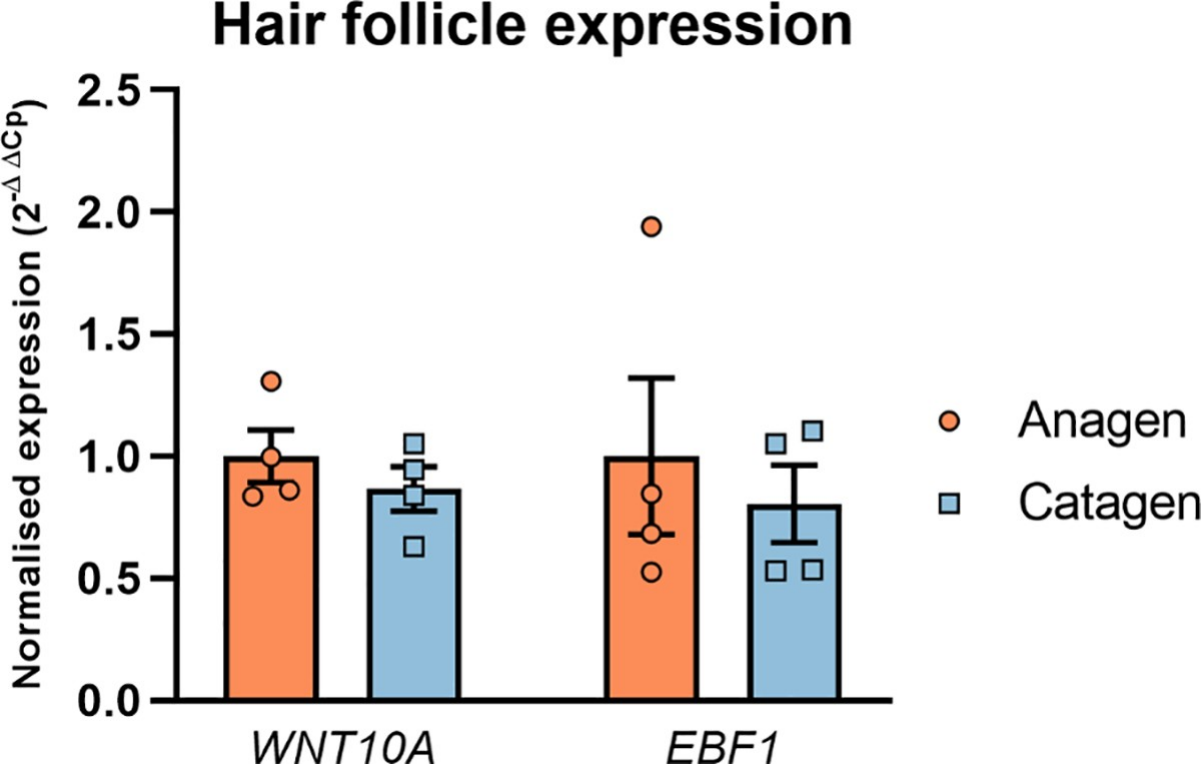
Results of the expression analysis for *WNT10A* and *EBF1* in microdissected human scalp hair follicles. The bar chart shows the expression values for *WNT10A* and *EBF1* in anagen and catagen hair follicles from four independent donors (12 hair follicles each). Normalization of the *WNT10A* and *EBF1* expression values were obtained using the measured expression levels for the housekeeping genes *ACTB* and *GAPDH*. The normalized expression values are shown relative to the gene expression in anagen hair follicles. The data show the mean values of three measurements ± SEM.

**Fig 4:**
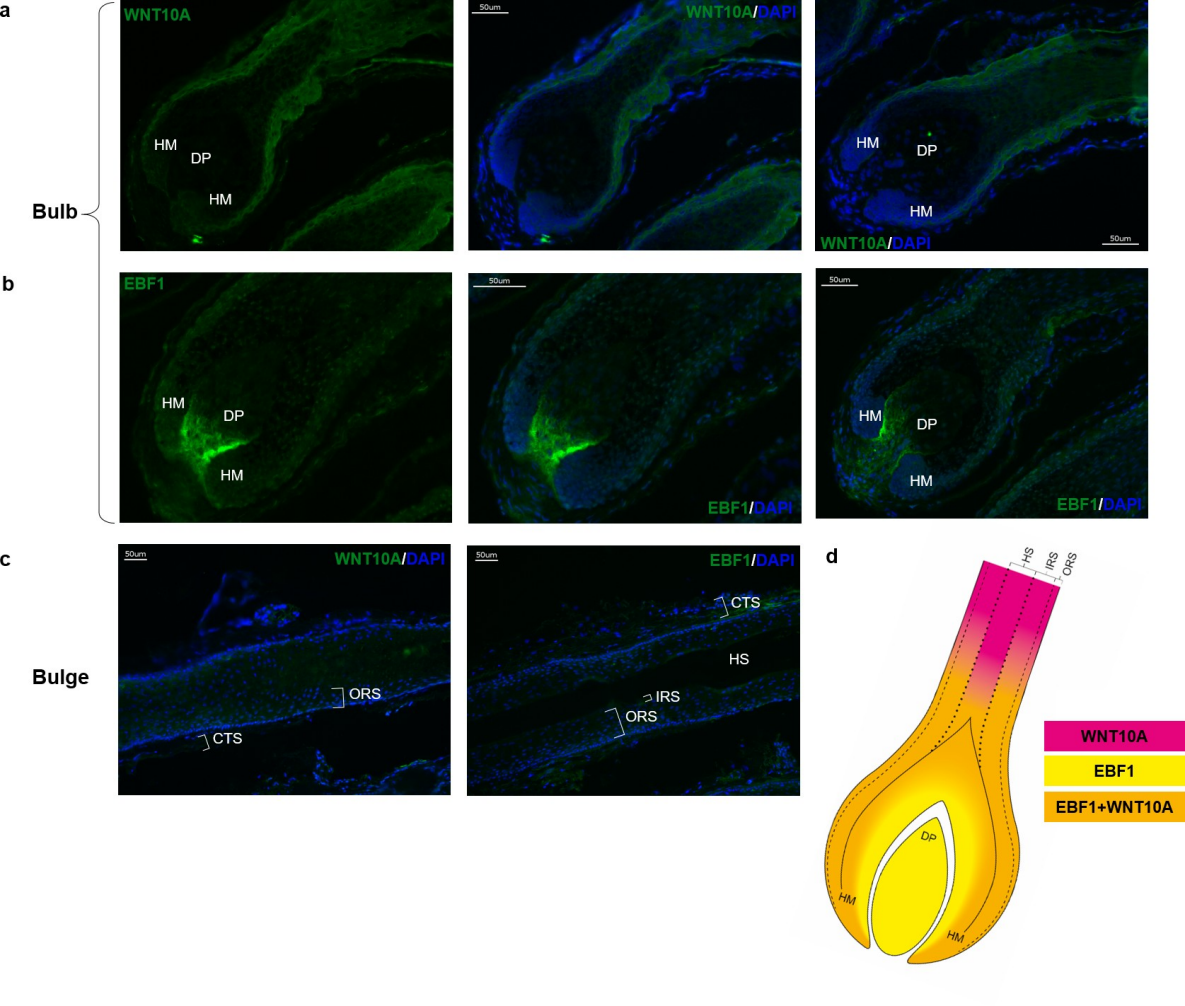
WNT10A and EBF1 expression in human scalp hair follicles. Representative images of WNT10A and EBF1 protein expression in microdissected human scalp hair follicles from three different donors (independent experiments). (A) Cytosolic expression of WNT10A (green, Alexa Fluor 488) in outer and inner root sheath and hair shaft keratinocytes. (B) Nucleic expression of EBF1 (green, Alexa FLuor 488) in the dermal papilla, hair matrix, hair shaft as well as outer and inner root sheath keratinocytes. (C) Expression of WNT10A and EBF1 in the hair follicle bulge area. Nuclear counterstaining for all images was performed with DAPI (blue). (D) Illustration of EBF1 (yellow) and WNT10A (pink) expression and co-localization (orange) in microdissected human anagen hair follicles. S3 Fig additionally provides a higher magnification. CTS—connective tissue sheath, DAPI—4′,6-diamidino-2-phenylindole, DP—dermal papilla, HM—hair matrix, HS—hair shaft, IRS—inner root sheath, ORS—outer root sheath. Original magnification ×200.

In addition to the analysis of a possible regulatory interaction in the *in vitro* model, the detection of co-expression of genes or their protein products in phenotypically relevant tissue can provide additional evidence for a functional interaction. Therefore, the expression of the candidate genes *WNT10A* and *EBF1* and their resulting protein products were investigated in microdissected human temporal scalp hair follicle specimen [18] using qRT-PCR and immunofluorescence analysis, respectively. Our analyses revealed co-expression of *WNT10A* and *EBF1* and their protein products in human anagen hair follicles (Figs 3 and 4). *WNT10A* and *EBF1* mRNA co-expression was also detected in human (early) catagen hair follicles. Here, the expression of both genes was slightly (~10%), but not significantly, reduced (Fig 3). No immunofluorescence data were available for catagen stage.

Cytosolic WNT10A expression was predominant in keratinocytes from the (proximal) outer and inner root sheath of the bulb area and the hair shaft, while no expression was observed in the dermal papilla (Fig 4A). EBF1 expression was detected in the nuclei of inner and outer root sheath, hair matrix, hair shaft and at a low level in the dermal papilla (Fig 4B and S3 Fig). In the bulge area of the hair shaft, WNT10A was expressed in the inner and outer root sheath, while no EBF1 expression was observed (Fig 4C). Co-localization of EBF1 and WNT10A expression is limited to the inner and outer root sheath and the hair shaft of the human hair follicle bulb area (Fig 4D) suggesting that the *in silico* and *in vitro* detected interaction of *WNT10A* and EBF1 occurs in these cells. Fluorescence images taken from two additional independent donors confirmed these observations (S3 Fig).

## Discussion

An improved understanding of the underlying molecular mechanisms at the level of individual and across risk loci is desirable in order to understand the exact pathomechanisms involved in the development of complex genetic diseases such as MPB. In this study, we aimed at elucidating the underlying molecular mechanism at the *WNT10A* locus on chr. 2q35. Our HaploReg database query mapped the 2q35 association signal to a binding site for EBF1, a transcription factor that has been highlighted as the likely candidate gene at a second MPB risk locus on chr. 5q33.3 [11]. Subsequent *in silico* analyses predicted an MPB risk allele dependent change in EBF1 binding affinity to its target site within *WNT10A*. These findings led us to hypothesize that the MPB risk loci on 2q35 and 5q33.3 are functionally connected through EBF1, which acts as a regulator of *WNT10A* expression in the control of hair growth cycling. Indeed, our experimental analyses using *in vitro* cell culture models and luciferase assays revealed that EBF1 is able to induce gene expression via the *WNT10A* promoter, where *WNT10A* promoter activation is positively correlated with EBF1 stimulation. These data suggest that EBF1 acts as a transcriptional activator of *WNT10A*, which is in accordance with previous studies, that showed that EBF1 acts mainly as a transcriptional activator in human and mouse B-cells [19]. Moreover, our data from HEK-293T cells lend experimental support for the HaploReg deposited protein-DNA interaction data retrieved from ChIP-seq analysis of lymphoblastoid cells (GM12878) [20], suggesting that EBF1 may be a regulator of *WNT10A* expression across different cell-types and tissues. Furthermore, we demonstrate that *WNT10A* promoter activation via EBF1 is dependent on the MPB risk variant rs3856551, where the risk allele (rs3856551-T) led to a significantly lower *WNT10A* promoter activation upon stimulation with EBF1 compared to the non-risk allele (rs3856551-C). These experimental data contradict the results of the *in silico* prediction of EBF1 binding affinity at 2q35 that suggested a slight increase in EBF1 binding affinity for the MPB risk allele (MSS [rs3856551-T] = 71% vs MSS [rs3856551-C] = 66%). However, the experimental data are in line with the assumption that EBF1 acts as a transcriptional activator of *WNT10A* expression and the previous mRNA expression data that revealed a reduced expression of *WNT10A* in hair follicles of 2q35 risk allele carriers [8]. The discrepancy between the *in silico* predicted and the experimental data once more highlights the value of experimental verification of *in silico* data in phenotypically relevant tissue.

The fact that expression of WNT10A and EBF1 was confirmed in microdissected human scalp hair follicles both on mRNA and protein level lends further support to our hypothesis that the observed regulatory interaction between EBF1 and *WNT10A* is of relevance to (healthy) hair biology. The slight reduction of *WNT10A*/*EBF1* expression that was observed between anagen and early catagen hair follicles, may point to a predominant relevance of *WNT10A*/EBF1 interaction during anagen. Immunohistochemical staining and subsequent immunofluorescence microscopy point to a co-localization of WNT10A/EBF1 expression in inner and outer root sheath and hair shaft within the hair follicle bulb area. The nucleolar localization of EBF1 and the cytoplasmic localization of WNT10A are in line with their function as a transcription factor and a signaling molecule, respectively. While this is the first study to perform a detailed investigation of the expression of EBF1 in human hair follicles, our immunohistochemical stainings for WNT10A support recent *in situ* hybridization data, that suggest a role for *WNT10A* in the regeneration of the outer root sheath during early anagen [21]. Intact cycling of the hair follicle is a complex procedure that requires constant reciprocal cross-talk between epithelial and mesenchymal compartments. Research suggests that epithelial outer root sheath cells are in steady exchange with mesenchymal dermal papilla cells to regulate hair growth [22]. Miscommunication among these hair follicle substructures is likely to result in changes in hair growth cycle dynamics, which are one of the characteristic pathophysiological signs in MPB [1].

**Fig 5:**
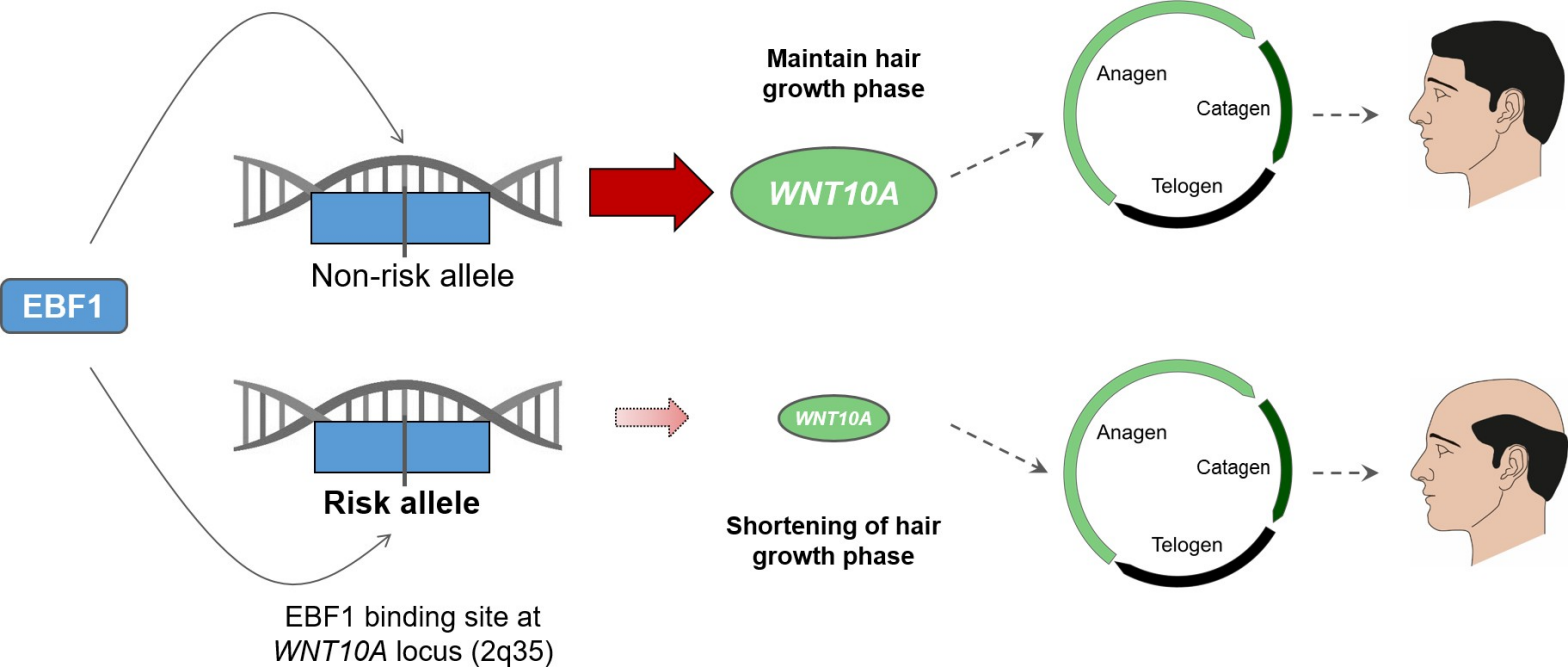
Illustration of the likely functional mechanism at the MPB 2q35 risk locus. Based on previous research that confirmed a role of WNT signaling in the initiation and maintenance of the hair growth phase (anagen) we suggest the following functional mechanism: EBF1 binds to its recognition sequence within the *WNT10A* gene (2q35) and activates *WNT10A* expression, thereby contributing to healthy hair cycling. The binding affinity of EBF1 to its target site at 2q35 is influenced by the allelic expression of the MPB associated variant rs3856551. In the presence of the MPB risk allele (rs3856551-T), there is a decreased binding affinity of EBF1 to its target sequence. This results in a reduced expression of *WNT10A* as compared to carriers of the non-risk allele (rs3856551-C) that eventually leads to the MPB typical changes in the initiation and maintenance of the anagen phase.

Our present data suggest that EBF1 and WNT10A are involved in this epithelial/mesenchymal crosstalk in the bulb area and that the MPB risk allele dependent decrease in EBF1 mediated *WNT10A*-promoter activation and *WNT10A* expression contributes to the MPB typical changes in hair cycle dynamics. Since we did not detect a co-localization of both proteins in the bulge area, which harbors the hair follicle stem cell population [23], we conclude that the observed regulatory interaction between EBF1 and *WNT10A* is more likely to contribute to the characteristic anagen shortening rather than to the delayed induction of a new hair growth cycle. Based on these findings, we propose the following extended model for the underlying functional mechanism at the 2q35 MPB risk locus: Upon binding to its recognition site at 2q35, the transcription factor EBF1 contributes to healthy hair cycling via transcriptional activation of *WNT10A*, which plays a role in anagen initiation and maintenance. Binding affinity of EBF1 to its 2q35 target sequence is impacted by the MPB risk variant rs3856551, where risk allele carriers (rs3856551-T) show a reduced binding affinity. This MPB associated reduction of EBF1 binding affinity in turn leads to a decrease in EBF1-mediated activation of *WNT10A* expression and contributes to a premature catagen entry and a prolongation of the latency phase before the re-entry into a new anagen phase (Fig 5). The shortening of the anagen phase and the prolongation of the latency period will eventually result in a reduced hair growth and a shift in the ratio of anagen to catagen hair follicles which is typical for MPB affected scalp.

In conclusion, this is the first functional follow-up study on MPB to show a regulatory interaction between candidate genes at two independent GWAS loci. Our data yield unprecedented insights into the underlying biological mechanism at the *WNT10A* locus on chr. 2q35 and suggest a role for EBF1-mediated *WNT10A* expression in the regulation and maintenance of the hair cycle growth phase.

Future in depth characterization of the regulatory interaction between *WNT10A*/EBF1 and identification of possible co-activators or repressors in the living hair follicle is warranted to broaden our understanding of their regulatory network in the human hair follicle. Moreover, systematic analyses of the relevance of transcriptional regulation through EBF1 in the development of MPB is desirable and may be addressed by systematic ChIP-Seq experiments in human hair follicles. In addition, investigation of the regulatory architecture of the 5q33.3 locus is warranted to enhance our understanding of the *WNT10A*/EBF1 regulatory network in MPB.

Eventually, these data will aid to bridge the gap between association finding and underlying biological mechanism and will lead to a deeper understanding of hair (loss) biology.

## Materials and methods

### *In silico* analyses

The HaploReg v4.1 database (https://pubs.broadinstitute.org/mammals/haploreg/haploreg.php, accessed 27 September 2019) was queried for functional annotation of rs7349332 and SNPs in LD (r^2^>0.8; 1000G Phase 1 European population [EUR]). Prediction of a potential *WNT10A* promoter region was performed by analyzing a five kb region upstream of the *WNT10A* start codon (chr2: 219,745,255, hg19) using the web-based tools Eponine [24], Promoter2.0 [25], CpGPlot [26], EVOPRINTER [27], phyloP [28, 29], MPromDb [30] and PoSSuMsearch [31]. PoSSuMsearch was also used to identify position and conservation of potential EBF1 binding sites based on the TRANSFAC database [32, 33] within the annotated EBF1 peak from the ENCODE ChIP-seq data set on chr2: 21,9745,845–219,746,160 (hg19) [34]. To assess a differential binding capacity of EBF1 due to rs3856551 the ‘motif similarity score’ was computed for both alleles (C, T).

### Construction of luciferase reporter vectors

**Fig 6:**
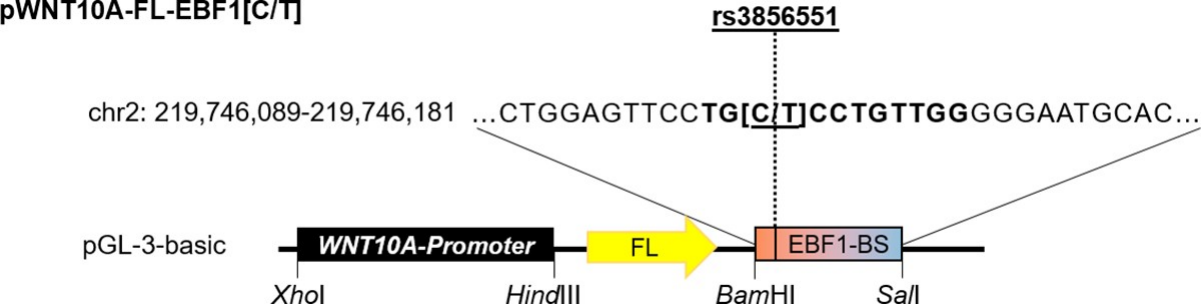
Schematic drawing of the reporter plasmid construct. The *WNT10A* promoter (1,641 bp) was cloned into pGL-3-basic using *Xho*I and *Hind*III. Using In-Fusion® HD-cloning the EBF1 binding site (EBF1-BS) (93 bp) containing the rs3856551 C- or T-allele was inserted between the *Bam*HI and *Sal*I restriction sites of pGL-3-basic.

The luciferase reporter plasmids (pWNT10A-EBF1[C/T]-FL) containing the putative *WNT10A* promoter and the EBF1 binding site including the MPB associated variant rs3856551, with either the C- or the T-allele, were constructed in two steps. First, a 1,641-bp region (chr2: 219,744,472–219,746,112 bp, hg19), predicted as potential core promoter upstream of the transcription start site of *WNT10A*, was amplified by PCR from human genomic DNA, digested with *Xho*I and *Hind*III, and cloned into the luciferase reporter vector pGL-3-basic (#E1751, Promega, Madison, WI, USA). For cloning of the EBF1 binding site comprising rs3856551-C/T down-stream of the *Firefly* luciferase gene, the In-Fusion® HD Cloning Plus Kit (#638910, TaKaRa, Takara Bio Inc., Kyoto, Japan) was used. Here, the EBF1 binding site (93 bp fragment, chr2: 219,746,089–219,746,181) was amplified from human genomic DNA as a template using the CloneAmp™ HiFi PCR Premix (#639298, Takara Bio Inc., Kyoto, Japan) and the respective In-Fusion® primers. The receiving plasmid was linearized by In-Fusion® PCR. Primer sequences and PCR conditions used for the respective cloning steps are listed in S2 Table. A schematic drawing of the constructed vector is depicted in Fig 6. Prof. Dr. R. Grosschedl (Max Planck Institute for Immunology and Epigenetics, Freiburg, Germany) kindly provided the EBF1 expression vector (pCMV-EBF1).

### Transient transfections

HEK-293T cells (#CRL-11268, obtained from ATCC, Manassas, VA, USA; regularly examined for *Mycoplasma* contamination using Venor®GeM Advance, Minerva Biolabs, Berlin Germany) were grown in DMEM (Lonza, Basel, Switzerland) supplemented with 10% *vol*/*vol* fetal bovine serum (Gibco, Thermo Fisher Scientific, Waltham, MA, USA) at 37°C in an atmosphere of 5% CO_2_. In 48-well plates, 5.2×10^4^ cells per well were seeded in 250 μl DMEM 24 h prior to transient transfection. Transfection was carried out using Lipofectamine® 2000 (Thermo Fisher Scientific, Waltham, MA, USA) according to the manufacturer’s protocol. In brief, for each well of the 48-well plate 25 μl OptiMEM (Gibco, Thermo Fisher Scientific, Waltham, MA, USA) was mixed with 0.5 μl Lipofectamine, 5 ng pRL-CMV (Promega, Madison, WI, USA), 50 ng reporter plasmid and 5–20 ng of pCMV5 or 5–20 ng pCMV-EBF1. The mixture was incubated for 5 min at room temperature and then added to the appropriate wells. Transfected cells were grown for 48 h at 37°C and 5% CO_2_ level and afterwards used for luciferase reporter assays. Each transfection was carried out in triplicate (technical replicates) in at least three independent experiments (biological replicates).

### Luciferase reporter assays

Forty-eight hours post-transfection, cells were carefully washed with 1×PBS (Lonza, Basel, Switzerland) and the Dual-Luciferase® Reporter Assay System (Promega, Madison, WI, USA) was used according to the manufactures’ protocol to measure *Firefly* and *Renilla* luciferase activity on a GloMax® Luminometer (Promega, Madison, WI, USA). Each measurement was done in at least three independent experiments. Since the addition of the EBF1 expression vector led to a significant decrease in *Renilla* luminescence in the present experiments (S2F–S2J Fig) it could not be used for normalization. Therefore, the results are given as non-normalized *Firefly* luminescence units (LU, if not otherwise indicated) and were directly equated to the activity of the examined *WNT10A* promoter.

### Tissue collection

Human hair follicles from temporal “clinically healthy” scalp skin were obtained from patients during routine face-lift surgery. For the immunofluorescence analyses, anagen hair follicles derived from three different individuals were microdissected [18] one day after surgery. Always three hair follicles were embedded in Shandon™ Cryomatrix™ (Thermo Fisher Scientific, Waltham, MA, USA), snap-frozen in liquid nitrogen and stored at -80°C. The samples were cut into 6 μm cryosections and stored at -80°C until immunohistocemical staining. For the gene expression analyses 12 anagen and 12 early catagen hair follicles derived from four donors (biological replicates) each were microdissected one day after surgery (i.e. after overnight transport from collaborating surgeons), immediately immersed in 500 μl RNAlater™ (Thermo Fisher Scientific, Waltham, MA, USA) and stored at 4°C until RNA extraction. All experiments on human tissue were performed according to Helsinki guidelines and samples were collected after written patient consent. The studies were approved by the ethics committee of the University of Münster (approval number 2015-602-f-S) and the University of Bradford Ethical Tissue Bank (an ethically approved human research tissue bank, licensed by the Human Tissue Authority (HTA), Licence number: 12191) with approval from the National Research Ethics Service (NRES) Committee Yorkshire & The Humber—Leeds East (approval number 17/YH/0086).

### RNA extraction and real-time reverse transcription-polymerase chain reaction (qRT-PCR)

Total RNA from 12 human scalp hair follicles (anagen and early catagen stadium) of four donors (biological replicates) each was isolated using the RNeasy Mini Kit (Qiagen, Hilden, Germany) according to the manufactures’ instructions. 500 ng of RNA were reverse transcribed into cDNA using the Tetro™ cDNA Synthesis Kit (Bioline GmbH, Luckenwalde, Germany). For the gene expression assay 0.5 μl cDNA, 5 μl TaqMan® Fast Advanced Master Mix (Applied Biosystems, Foster City, CA, USA), 0.5 μl TaqMan® Gene Expression Assay (*WNT10A*: Hs00228741_m1, *EBF1*: Hs01092694_m1, *GAPDH*: Hs02786624_g1, *ACTB*: Hs01060665_g1, all Applied Biosystems, Foster City, CA, USA) were mixed. The samples were analyzed in triplicates (technical replicates) on a QuantStudio 3 Real-Time PCR System (Applied Biosystems, Waltham, MA, USA) using the following conditions: 95°C for 10 min, followed by 45 cycles of 95°C for 15 s and 60°C for 1 min. Relative expression of *WNT10A* and *EBF1* in relation to the housekeeping genes *GAPDH* and *ACTB* was calculated based on the calculated Δ-C_p_ values using the 2^-ΔΔ^Cp-method [35].

### Statistical analyses

All data are expressed as mean ±SEM. To evaluate statistical differences in luciferase assays, two-way analysis of variance (ANOVA) followed by Tuckey’s post-hoc test was applied to account for multiple comparisons. Given the exploratory design and moderate statistical power of this study, no correction for multiple testing was performed. For the statistical analysis of the qPCR results, the mean ±SEM of the measurements were calculated. *P*-values were assessed using a Student’s *t*-test for paired samples. *P*-values <0.05 were considered as statistically significant in all analyses. All calculations were performed within GraphPad Prism software version 9 (GraphPad Software Inc., San Diego, CA).

### Immunohistochemical staining and fluorescence microscopy

Immunohistochemical staining was used to analyze the expression pattern and localization of WNT10A and EBF1 in the human hair follicle. The cryosections were first air dried for 10 min at room temperature and then fixed for 10 min in acetone at -20°C (WNT10A) or 4% paraformaldehyde at room temperature (EBF1). After air drying for 10 min at room temperature the slides were washed three times for 5 min in wash buffer (WNT10A: Tris-buffered saline [TBS], EBF1: phosphate-buffered saline [PBS]). Cryosections for the WNT10A staining were blocked at room temperature for 60 min with a mixture of 20% normal goat serum, 1% bovine serum albumin (BSA) and 0.1% Tween20 in TBS. The WNT10A primary antibody (#ab62051, Abcam, Cambridge, UK) diluted 1:25 in TBS was applied overnight at 4° C. After washing the sections three times for 5 min in TBS, the sections were stained with a goat anti-rabbit IgG secondary antibody conjugated with Alexa Fluor 488 (1:200 in TBS, #A32731, Thermo Fisher Scientific, Waltham, MA, USA) for 45 min at room temperature. Cryosections for the EBF1 staining were pre-incubated at room temperature for 30 min with a mixture of 0.1% Triton-X, 1% BSA in PBS. The EBF1 primary antibody (#HPA061169, Sigma-Aldrich, St. Louis, MO, USA) diluted 1:20 in 1% BSA/PBS was applied overnight at 4°C. After washing the cryosections three times for 5 min in PBS, the sections were stained for 45 min with a goat anti-rabbit IgG secondary antibody conjugated with fluorescein (1:400 in 1% BSA/PBS, #111-095-003, Jackson Research Laboratories, West Grove, PA, USA). To enhance the fluorescence signal the sections were incubated with a tertiary antibody directed against fluorescein conjugated to Alexa Fluor 488 (1:400 in 1% BSA/PBS, #A-11090, Invitrogen, Waltham, MA, USA) for 30 min at room temperature. After washing the cryosections three times for 5 min in their respective wash buffer, the sections were counterstained with DAPI (1 μg/ml, Boehringer Mannheim, Mannheim, Germany) for 1 min, washed three times for 5 min in their wash buffer and mounted with Fluoromount-G™ (Southern Biotechnologies, Birmingham, AL, USA). For all immunostainings primary antibodies were omitted for negative controls. The sections were dried for 30 min at room temperature and stored at -20°C until fluorescence microscopy. For fluorescence microscopy and photographs a BZ-9000 (Keyence, Osaka, Japan) fluorescence microscope maintaining a constant set exposure time throughout imaging was used.

## Supporting information

S1 Fig Schematic representation of the study design and workflow. Schematic representation of the study background, the key scientifc questions, the experimental workflow, and the key findings and conclusion of the present study.
(TIF)

S2 Fig Results of luciferase assays for pWNT10A-FL-EBF1[C/T]. (A-E) Activity of *WNT10A* promoter with respect to EBF1 and rs3856551 in five separate experiments. (F-J) *Renilla* (RL) values of the five luciferase assays. The number in brackets marks correlating experiemnts. B and G belong to Fig 3 in the main manuscript.
(TIF)

S3 Fig Representative pictures of WNT10A (A) and EBF1 (B, C) stainings in hair follicle samples from two additional independent individuals. For (C) Alexa Fluor 555 (1:200 in PBS, 37°C for 1 h, #A32794, Invitrogen) was used as the secondary antibody. Orignal magificaiton x 200.
(TIF)

S1 Table Raw Firefly (FL) and Renilla (RL) values of the performed luciferase assays. (DOCX)

S2 Table Overview of primer sequences, template DNA and PCR conditions used for cloning. (DOCX)

## References

[1] Heilmann-Heimbach S, Hochfeld LM, Paus R, Nöthen MM. Hunting the genes in male-pattern alopecia: how important are they, how close are we and what will they tell us? Exp Dermatol. 2016;25: 251–7. doi: 10.1111/exd.12965 26843402

[2] Nyholt DR, Gillespie NA, Heath AC, Martin NG. Genetic Basis of Male Pattern Baldness. J Invest Dermatol. 2003;121: 1561–4. doi: 10.1111/j.1523-1747.2003.12615.x 14675213

[3] Yap CX, Sidorenko J, Wu Y, Kemper KE, Yang J, Wray NR, et al. Dissection of genetic variation and evidence for pleiotropy in male pattern baldness. Nat Commun. 2018; 1–12. doi: 10.1038/s41467-017-02088-w 30573740 PMC6302097

[4] Richards JB, Yuan X, Geller F, Waterworth D, Bataille V, Glass D, et al. Male-pattern baldness susceptibility locus at 20p11. Nat Genet. 2008;40: 1282–4. doi: 10.1038/ng.255 18849991 PMC2672151

[5] Hillmer AM, Brockschmidt FF, Hanneken S, Eigelshoven S, Steffens M, Flaquer A, et al. Susceptibility variants for male-pattern baldness on chromosome 20p11. Nat Genet. 2008;40: 1279–81. doi: 10.1038/ng.228 18849994

[6] Brockschmidt FF, Heilmann S, Ellis JA, Eigelshoven S, Hanneken S, Herold C, et al. Susceptibility variants on chromosome 7p21.1 suggest HDAC9 as a new candidate gene for male-pattern baldness. Br J Dermatol. 2011;165: 1293–1302. doi: 10.1111/j.1365-2133.2011.10708.x 22032556

[7] Li R, Brockschmidt FF, Kiefer AK, Stefansson H, Nyholt DR, Song K, et al. Six novel susceptibility Loci for early-onset androgenetic alopecia and their unexpected association with common diseases. PLoS Genet. 2012;8: e1002746. doi: 10.1371/journal.pgen.1002746 22693459 PMC3364959

[8] Heilmann S, Kiefer AK, Fricker N, Drichel D, Hillmer AM, Herold C, et al. Androgenetic alopecia: identification of four genetic risk loci and evidence for the contribution of WNT signaling to its etiology. J Invest Dermatol. 2013;133: 1489–96. doi: 10.1038/jid.2013.43 23358095

[9] Pickrell JK, Berisa T, Liu JZ, Ségurel L, Tung JY, Hinds DA. Detection and interpretation of shared genetic influences on 42 human traits. Nat Genet. 2016;48: 709–17. doi: 10.1038/ng.3570 27182965 PMC5207801

[10] Hagenaars SP, Hill WD, Harris SE, Ritchie SJ, Davies G, Liewald DC, et al. Genetic prediction of male pattern baldness. Noethen MM, editor. PLoS Genet. 2017;13: e1006594. doi: 10.1371/journal.pgen.1006594 28196072 PMC5308812

[11] Heilmann-Heimbach S, Herold C, Hochfeld LM, Hillmer AM, Nyholt DR, Hecker J, et al. Meta-analysis identifies novel risk loci and yields systematic insights into the biology of male-pattern baldness. Nat Commun. 2017;8: 14694. doi: 10.1038/ncomms14694 28272467 PMC5344973

[12] Pirastu N, Joshi PK, de Vries PS, Cornelis MC, McKeigue PM, Keum N, et al. GWAS for male-pattern baldness identifies 71 susceptibility loci explaining 38% of the risk. Nat Commun. 2017;8: 1584. doi: 10.1038/s41467-017-01490-8 29146897 PMC5691155

[13] ENCODE Project Consortium. An integrated encyclopedia of DNA elements in the human genome. Nature. 2012;489: 57–74. doi: 10.1038/nature11247 22955616 PMC3439153

[14] Lappalainen T. Functional genomics bridges the gap between quantitative genetics and molecular biology. Genome Res. 2015;25: 1427–31. doi: 10.1101/gr.190983.115 26430152 PMC4579327

[15] McGuire AL, Gabriel S, Tishkoff SA, Wonkam A, Chakravarti A, Furlong EEM, et al. The road ahead in genetics and genomics. Nat Rev Genet. 2020;21: 581–596. doi: 10.1038/s41576-020-0272-6 32839576 PMC7444682

[16] Whiting DA. Diagnostic and predictive value of horizontal sections of scalp biopsy specimens in male pattern androgenetic alopecia. J Am Acad Dermatol. 1993;28: 755–63. doi: 10.1016/0190-9622(93)70106-4 8496421

[17] Kaufman KD. Androgens and alopecia. Mol Cell Endocrinol. 2002;198: 89–95. doi: 10.1016/s0303-7207(02)00372-6 12573818

[18] Langan EA, Philpott MP, Kloepper JE, Paus R. Human hair follicle organ culture: theory, application and perspectives. Exp Dermatol. 2015;24: 903–11. doi: 10.1111/exd.12836 26284830

[19] Gisler R, Jacobsen SE, Sigvardsson M. Cloning of human early B-cell factor and identification of target genes suggest a conserved role in B-cell development in man and mouse. Blood. 2000;96: 1457–64. 10942392

[20] Wang J, Zhuang J, Iyer S, Lin X-Y, Greven MC, Kim B-H, et al. Factorbook.org. Nucleic Acids Res. 2013;41: D171–D176. doi: 10.1093/nar/gks1221 23203885 PMC3531197

[21] Hawkshaw NJ, Hardman JA, Alam M, Jimenez F, Paus R. Deciphering the molecular morphology of the human hair cycle: Wnt signalling during the telogen-anagen transformation. Br J Dermatol. 2019; 0–2. doi: 10.1111/bjd.18356 31314901

[22] Ohn J, Kim KH, Kwon O. Evaluating hair growth promoting effects of candidate substance: A review of research methods. J Dermatol Sci. 2019;93: 144–149. doi: 10.1016/j.jdermsci.2019.02.004 30904351

[23] Rishikaysh P, Dev K, Diaz D, Qureshi W, Filip S, Mokry J. Signaling Involved in Hair Follicle Morphogenesis and Development. Int J Mol Sci. 2014;15: 1647–1670. doi: 10.3390/ijms15011647 24451143 PMC3907891

[24] Down TA, Hubbard TJP. Computational detection and location of transcription start sites in mammalian genomic DNA. Genome Res. 2002;12: 458–61. doi: 10.1101/gr.216102 11875034 PMC155284

[25] Knudsen S. Promoter2.0: for the recognition of PolII promoter sequences. Bioinformatics. 1999;15: 356–61. doi: 10.1093/bioinformatics/15.5.356 10366655

[26] Rice P, Longden I, Bleasby A. EMBOSS: the European Molecular Biology Open Software Suite. Trends Genet. 2000;16: 276–7. doi: 10.1016/s0168-9525(00)02024-2 10827456

[27] Odenwald WF, Rasband W, Kuzin A, Brody T. EVOPRINTER, a multigenomic comparative tool for rapid identification of functionally important DNA. Proc Natl Acad Sci U S A. 2005;102: 14700–5. doi: 10.1073/pnas.0506915102 16203978 PMC1239946

[28] Siepel A, Bejerano G, Pedersen JS, Hinrichs AS, Hou M, Rosenbloom K, et al. Evolutionarily conserved elements in vertebrate, insect, worm, and yeast genomes. Genome Res. 2005;15: 1034–50. doi: 10.1101/gr.3715005 16024819 PMC1182216

[29] Pollard KS, Hubisz MJ, Rosenbloom KR, Siepel A. Detection of nonneutral substitution rates on mammalian phylogenies. Genome Res. 2010;20: 110–21. doi: 10.1101/gr.097857.109 19858363 PMC2798823

[30] Sun H, Palaniswamy SK, Pohar TT, Jin VX, Huang TH-M, Davuluri R V. MPromDb: an integrated resource for annotation and visualization of mammalian gene promoters and ChIP-chip experimental data. Nucleic Acids Res. 2006;34: D98–103. doi: 10.1093/nar/gkj096 16381984 PMC1347458

[31] Beckstette M, Homann R, Giegerich R, Kurtz S. Fast index based algorithms and software for matching position specific scoring matrices. BMC Bioinformatics. 2006;7: 389. doi: 10.1186/1471-2105-7-389 16930469 PMC1635428

[32] Wingender E, Chen X, Hehl R, Karas H, Liebich I, Matys V, et al. TRANSFAC: an integrated system for gene expression regulation. Nucleic Acids Res. 2000;28: 316–9. doi: 10.1093/nar/28.1.316 10592259 PMC102445

[33] Matys V, Fricke E, Geffers R, Gössling E, Haubrock M, Hehl R, et al. TRANSFAC: transcriptional regulation, from patterns to profiles. Nucleic Acids Res. 2003;31: 374–8. doi: 10.1093/nar/gkg108 12520026 PMC165555

[34] Wang J, Zhuang J, Iyer S, Lin X-Y, Greven MC, Kim B-H, et al. Factorbook.org: a Wiki-based database for transcription factor-binding data generated by the ENCODE consortium. Nucleic Acids Res. 2013;41: D171–D176. doi: 10.1093/nar/gks1221 23203885 PMC3531197

[35] Livak KJ, Schmittgen TD. Analysis of relative gene expression data using real-time quantitative PCR and the 2(-Delta Delta C(T)) Method. Methods. 2001;25: 402–8. doi: 10.1006/meth.2001.1262 11846609

