# Supplementary figures and images for: Evidence for a functional interaction of WNT10A and EBF1 in male-pattern baldness
Source: PLoS One. 2021 Sep 10;16(9):e0256846. doi: 10.1371/journal.pone.0256846 (PMC8432770; doi:10.1371/journal.pone.0256846)

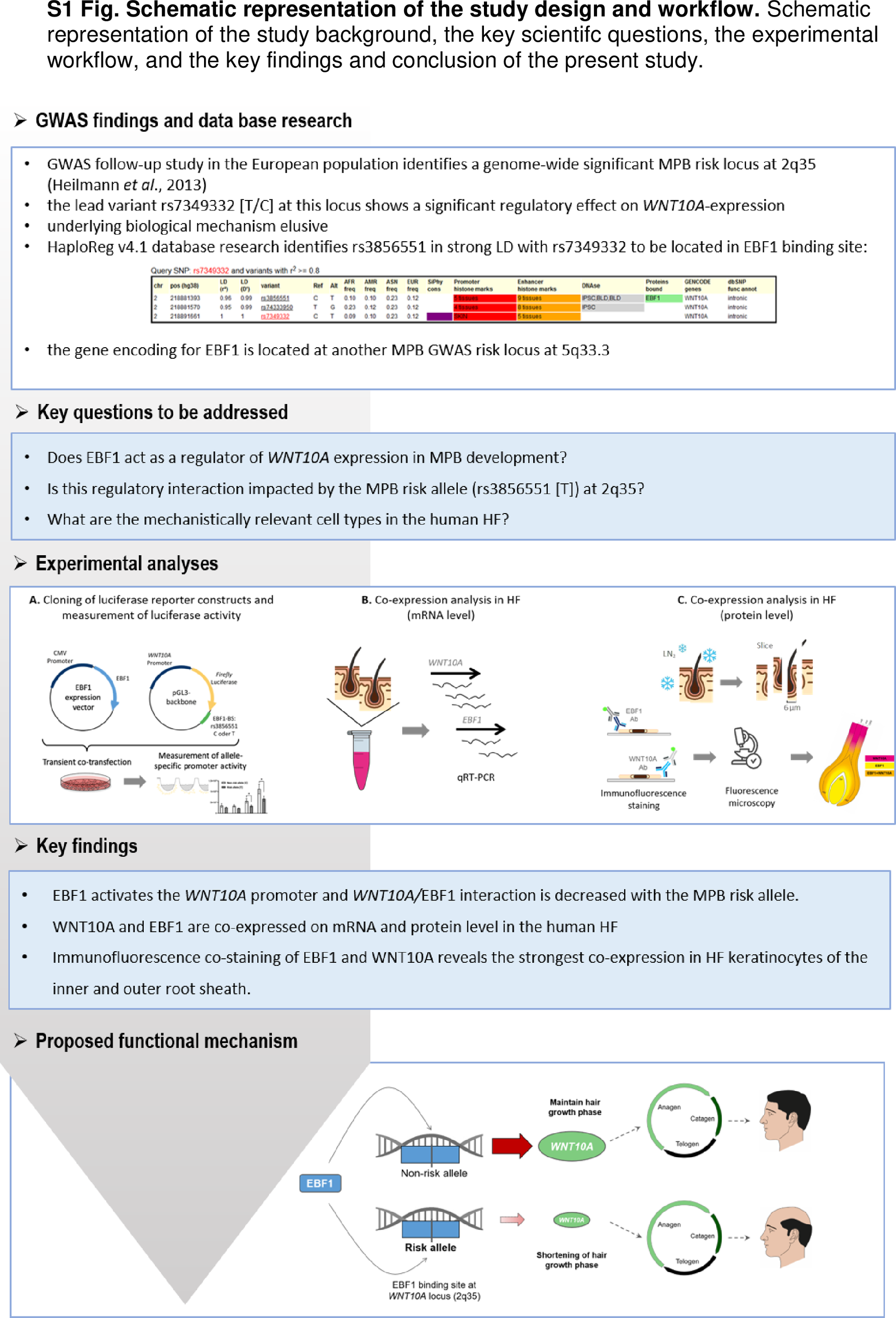

Supplement: S1 Fig — Schematic representation of the study background, the key scientifc questions, the experimental workflow, and the key findings and conclusion of the present study. (TIF) [file pone.0256846.s001.tif]

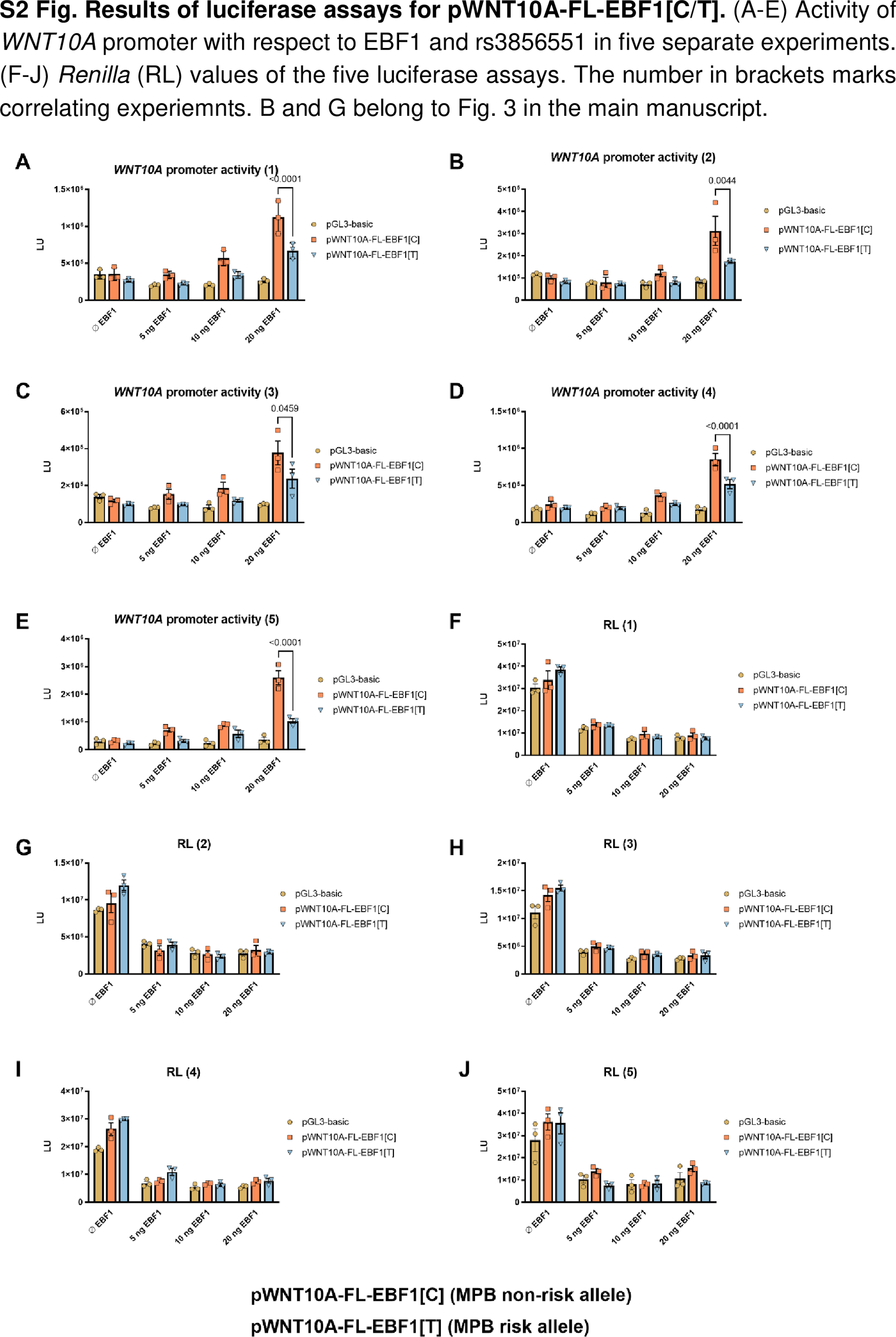

Supplement: S2 Fig — (A-E) Activity of WNT10A promoter with respect to EBF1 and rs3856551 in five separate experiments. (F-J) Renilla (RL) values of the five luciferase assays. The number in brackets marks correlating experiemnts. B and G belong to Fig 3 in the main manuscript. (TIF) [file pone.0256846.s002.tif]

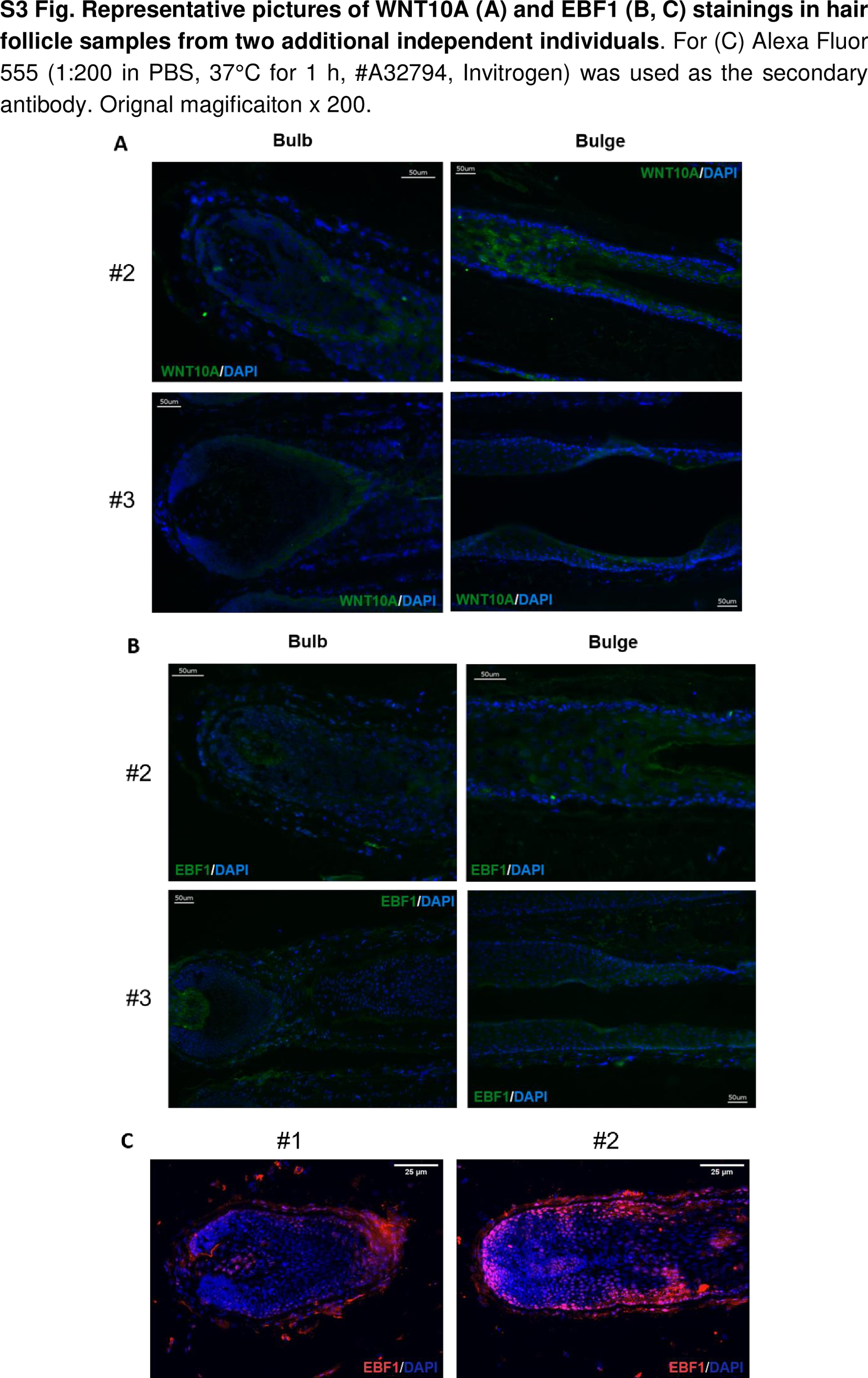

Supplement: S3 Fig — Representative pictures of WNT10A (A) and EBF1 (B, C) stainings in hair follicle samples from two additional independent individuals. For (C) Alexa Fluor 555 (1:200 in PBS, 37°C for 1 h, #A32794, Invitrogen) was used as the secondary antibody. Orignal magificaiton x 200. (TIF) [file pone.0256846.s003.tif]

**S2 Table. Overview of primer sequences, template DNA and PCR conditions used for cloning.**


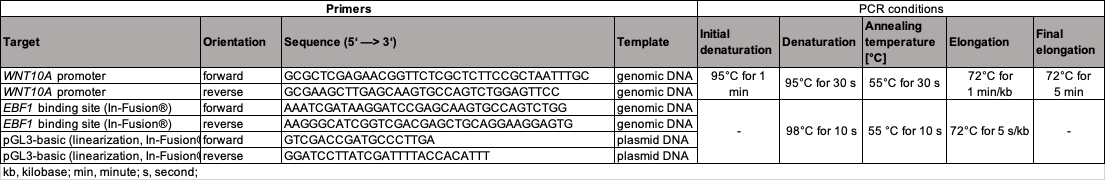

Supplement: S2 Table — (DOCX) [file pone.0256846.s005.docx]
