# Supplementary material for: Evidence for a functional interaction of WNT10A and EBF1 in male-pattern baldness
Source: PLoS One. 2021 Sep 10;16(9):e0256846. doi: 10.1371/journal.pone.0256846 (PMC8432770; doi:10.1371/journal.pone.0256846)
Supplement: S1 Table — (DOCX) [file pone.0256846.s004.docx]

**Table S1. Raw *Firefly* (FL) and *Renilla* (RL) values of the performed luciferase assays.**
